# Cardiovascular manifestations and outcomes in patients with leptospirosis admitted to a tertiary care center in the Coastal Karnataka region in India

**DOI:** 10.1007/s15010-026-02806-x

**Published:** 2026-05-04

**Authors:** M. Sudhakar Rao, Jyothi Samanth, Vidya Nayak, R. Padmakumar, Asmitha G. Nayak, P. K. Sanjana Nambiar, Shephali Rajesh Rao, Pratheeksha Rao

**Affiliations:** 1 Department of Cardiology, Manipal Hospital, Bangalore, Karnataka India; 2 Department of Cardiovascular Technology, Manipal College of Health Professions, Manipal Academy of Higher Education, Manipal, 576104 Karnataka India; 3 Department of Cardiology, Kasturba Medical College, Manipal, Manipal Academy of Higher Education, Manipal, Karnataka India

**Keywords:** Leptospirosis, Myocarditis, Heart failure, LV dysfunction

## Abstract

**Introduction:**

Leptospirosis is a globally prevalent zoonotic infection caused by Leptospira species. Cardiac involvement is increasingly recognized, yet large-scale studies comprehensively evaluating electrocardiographic and echocardiographic manifestations, including myocarditis, remain limited.

**Methods:**

This retrospective study included adult patients with clinically suspected leptospirosis and positive IgM ELISA admitted to a tertiary care hospital in South India between January 2016 and September 2020. Clinical features, electrocardiographic (ECG) findings, two-dimensional echocardiographic parameters, and in-hospital outcomes were analyzed. Binomial logistic regression was performed to identify independent predictors of all-cause mortality and myocarditis.

**Results:**

A total of 510 patients were analyzed (mean age 48.4 ± 13.7 years; 66.5% male). The mean hospital stay was 10.1 ± 6.1 days, with 40% requiring hospitalization beyond 10 days. Diabetes mellitus and hypertension were present in 16.7% and 16.9% of patients, respectively. Acute respiratory distress syndrome occurred in 10.6%, and 28% developed multiorgan dysfunction syndrome; 17.8% required hemodialysis. The most common ECG abnormalities were corrected QT interval prolongation (21.0%) and sinus tachycardia (20.8%). Echocardiographic evidence of myocarditis was observed in 10.4% of patients, including isolated left ventricular dysfunction (4.1%), isolated right ventricular dysfunction (3.9%), and biventricular dysfunction (2.4%). The overall in-hospital mortality rate was 8%. Increasing age, sinus tachycardia, QTc prolongation, hypertension, hemodialysis requirement, and myocarditis were independent predictors of mortality.

**Conclusions:**

Myocarditis represents a significant cardiac complication in leptospirosis and is associated with increased mortality. Routine cardiac evaluation may facilitate early detection and risk stratification.

**Clinical trial registration:**

Not applicable, as this study was retrospective in nature.

**Graphical abstract:**

**Figure:**
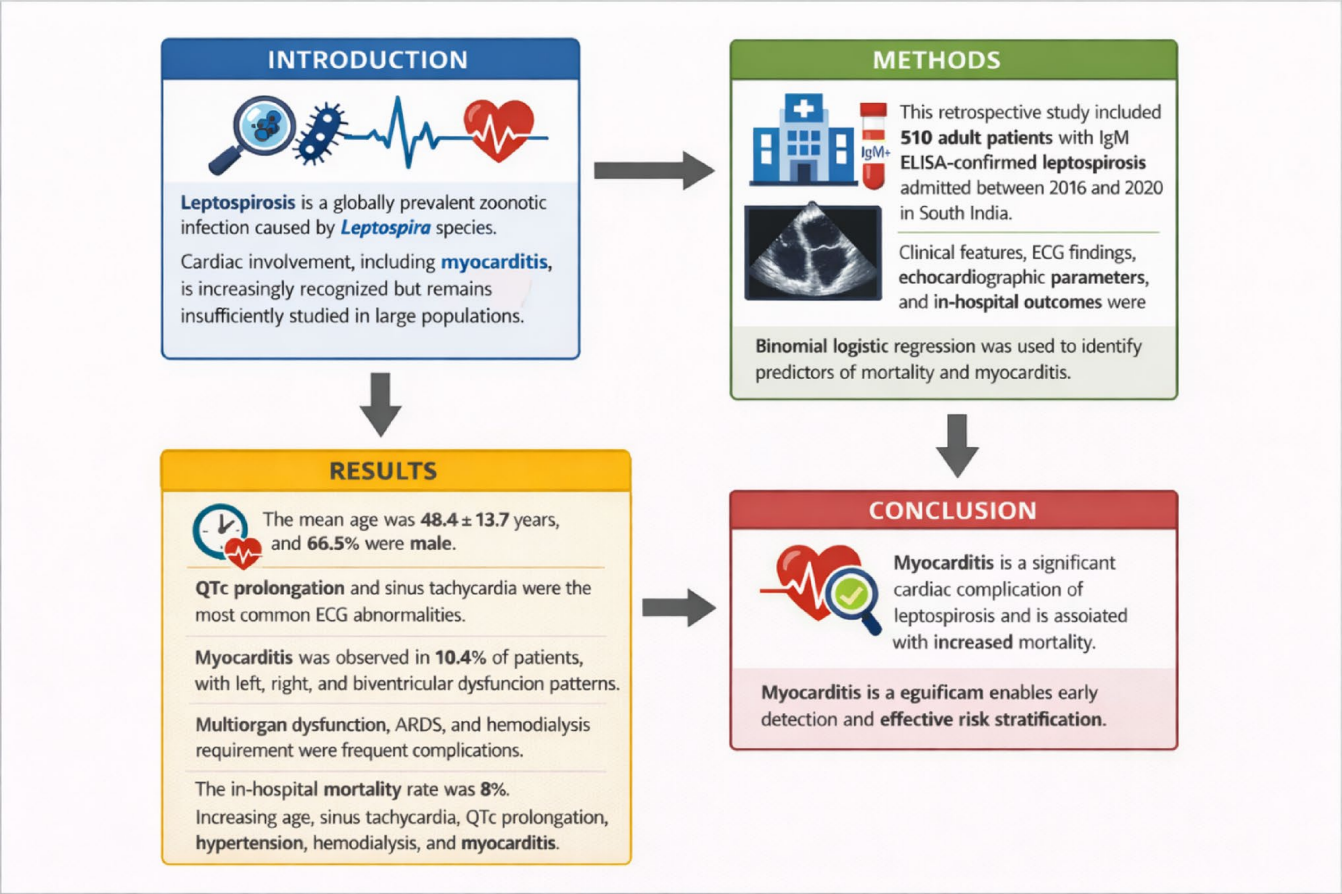

## Introduction

Leptospirosis is a globally prevalent animal-borne infectious disease that is caused by the spirochete bacterium Leptospira [1]. It is seen as strongly affecting tropical and semitropical regions with heavy rainfall, flooding and poor sanitation [2]. The disease was first reported in 1886 by physician Adolf Weil, Germany. In ancient China, the disease was described as an occupational hazard of rice harvesting [3]. The Japanese name for this disease is akiyami or autumn fever [3, 4]. In common language, it is popularly known as rat fever. Leptospirosis is a common and important infectious disease worldwide, causing more than 1 million cases and nearly 60,000 deaths each year. In Southeast Asia alone, approximately 270,000 cases and 14,000 deaths occur each year. In developing countries, the incidence ranges from 10 to 100 cases per 100,000 people annually. In India, the estimated annual burden ranges between 0.1 and 1 million cases, although fewer than 10,000 cases are formally reported. More than 500 cases are reported per year in Maharashtra, Tamil Nadu, Gujarat, and Kerala, and sporadic cases are recorded across several other states. Owing to limited awareness and inadequate laboratory diagnostic facilities, leptospirosis is often missed and inadequately documented in India [5]. It places a significant burden on low socioeconomic groups, causing high rates of morbidity and death. Infection can occur through direct or indirect contact with animal urine or from water or soil contaminated by infected animal urine [2]. Rats are the most common carriers of pathogenic organisms and are the primary source of human infection because they live close to human settlements and shed large quantities of these organisms for prolonged periods. Other animals, including dogs and various domestic animals, can also carry and transmit the pathogen, although they contribute less significantly to human infection than rats do [1]. Veterinarians, animal healthcare personnel, sanitation workers, and individuals employed in agricultural environments are particularly vulnerable to acquiring this infection [5]. Leptospiral infection in humans presents with a broad clinical spectrum. It can range from a subclinical illness to severe multiorgan involvement [2]. Serious complications include acute kidney injury, hepatic dysfunction, myocardial involvement, and pulmonary hemorrhage [1]. The major target organs are the liver, lungs, and kidneys.

In the liver, disruption of hepatocyte junctions and apoptosis lead to bile leakage and jaundice. In the lungs, widespread alveolar hemorrhage may result in severe pulmonary hemorrhage syndrome. In the kidneys, tubular damage and interstitial nephritis cause renal dysfunction. This dysfunction can range from mild polyuria to acute renal failure, known as Weil’s syndrome [6]. Although several studies have demonstrated cardiac involvement in leptospirosis, including electrocardiographic abnormalities and myocardial injury, it is often underrecognized in clinical practice because of the absence of specific or overt cardiac symptoms [7]. Cardiac manifestations in leptospirosis include chest pain, refractory shock and pulmonary edema. Electrocardiography may reveal some nonspecific changes. These can include atrial fibrillation, AV blocks, arrhythmias, ventricular premature contractions, T-wave inversion, and ventricular repolarization abnormalities [8, 9]. Abnormal levels of body salts (electrolytes) may contribute to ECG changes. It is believed that Leptospira may directly affect the sodium (Na⁺), potassium (K⁺), and chloride (Cl⁻) transport systems in kidney tubules, leading to these electrolyte disturbances. Postmortem studies have clearly shown that inflammation of the heart muscle leads to a condition called myocarditis and inflammation of the blood vessels in patients with leptospirosis. However, the exact mechanism by which leptospirosis affects the heart remains unclear. When heart involvement is identified through either ECG changes or clinical symptoms, it is generally associated with a poor prognosis and a high risk of complications [10]. Data regarding cardiovascular complications and their determinants in patients with leptospirosis remain limited. This retrospective analysis evaluated the spectrum of cardiac manifestations, associated outcomes, and their predictors in individuals with leptospirosis admitted to a tertiary care hospital in coastal-Karnataka.

## Materials and methods

This retrospective analysis was carried out at a tertiary care center in South India. Prior to the commencement of this study, ethical clearance was obtained from the Institutional Ethics Committee of Kasturba Medical College and Kasturba Hospital (IEC No.: 125/2021). The study was conducted in accordance with the ethical standards of the Institutional Ethics Committee. As this was a retrospective study using anonymized patient data, the requirement for informed consent was waived by the Ethics Committee. The study included all patients admitted from January 2016 to September 2020 who satisfied the clinical diagnostic criteria for leptospirosis and who tested positive for IgM by ELISA. Only individuals aged 18 years or older were eligible. Patients with preexisting cardiac disorders, including ischemic heart disease, valvular abnormalities, congenital heart disease, cardiomyopathy, or previously documented arrhythmias, were excluded. Patients with mixed infections, including scrub typhus, dengue fever, septicemia confirmed by blood culture, enteric fever, or COVID-19, were also excluded. Patients were further excluded if comprehensive cardiac assessment data, including electrocardiographic and echocardiographic findings, were not available. Demographic details such as age and sex, along with associated comorbidities, including diabetes mellitus and hypertension, were documented. Baseline laboratory parameters comprising complete blood counts, renal and hepatic function tests, and cardiac biomarkers such as CK-MB and troponin-T (where available) were obtained and analysed. Cardiac troponin T levels were assessed using the institutional standard electrochemiluminescence assay in patients for whom testing was clinically indicated during admission. Chest X-ray findings were also reviewed to assess for evidence of acute respiratory distress syndrome.

A baseline electrocardiogram recorded at hospital admission as part of routine clinical evaluation was reviewed. ECG parameters, including heart rate, conduction abnormalities, rhythm disturbances, ST–T changes, corrected QT (QTc) interval, repolarization abnormalities, and features suggestive of pericarditis, were analyzed. Rhythm abnormalities such as atrial fibrillation and ventricular arrhythmia, such as ventricular tachycardia, occurring at any time during hospitalization were also documented from medical records. Sinus bradycardia and sinus tachycardia were defined as heart rates below 60 beats per minute and above 100 beats per minute, respectively. A corrected QT interval exceeding 460 ms in men and 480 ms in women was considered indicative of QTc prolongation.

Two-dimensional echocardiographic findings obtained during clinical assessment were analyzed. The evaluation included measurements of left ventricular end-diastolic and end-systolic dimensions and volumes, ejection fraction, fractional shortening, regional wall motion abnormalities, and global hypokinesia. Additional parameters assessed were mitral and tricuspid regurgitation, right ventricular systolic pressure, pericardial effusion, and the presence of isolated left ventricular dysfunction, isolated right ventricular dysfunction, or combined biventricular dysfunction. A right ventricular systolic pressure greater than 50 mmHg was considered indicative of significant pulmonary hypertension.

Myocarditis was defined as follows:

- Presence of signs and symptoms consistent with heart failure along with newly identified left or right ventricular dysfunction in patients who had a normal echocardiogram at the time of admission.
- Clinical features of heart failure with associated left ventricular dysfunction, along with biochemical markers indicating myocardial injury (troponin > 0.1).

Isolated left ventricular dysfunction was considered present when the ejection fraction was less than 50%, irrespective of whether troponin T levels were elevated.

Isolated right ventricular dysfunction was defined as evidence of right atrial or right ventricular enlargement, low-pressure tricuspid regurgitation, and/or a tricuspid annular plane systolic excursion (TAPSE) measuring less than 16 mm.

The primary outcome measures included all-cause in-hospital mortality and the occurrence of myocarditis. Additional outcomes, such as the need for hemodialysis, duration of hospital stay, and development of multiorgan dysfunction syndrome, were also recorded.

Statistical analysis was carried out using SPSS software. Descriptive statistics were used to summarize proportions, means, and standard deviations. Associations between two categorical variables were evaluated using the chi-square test. Multivariate analysis was conducted to determine independent predictors of outcomes, including myocarditis and overall in-hospital mortality.

## Results

**Fig. 1:**
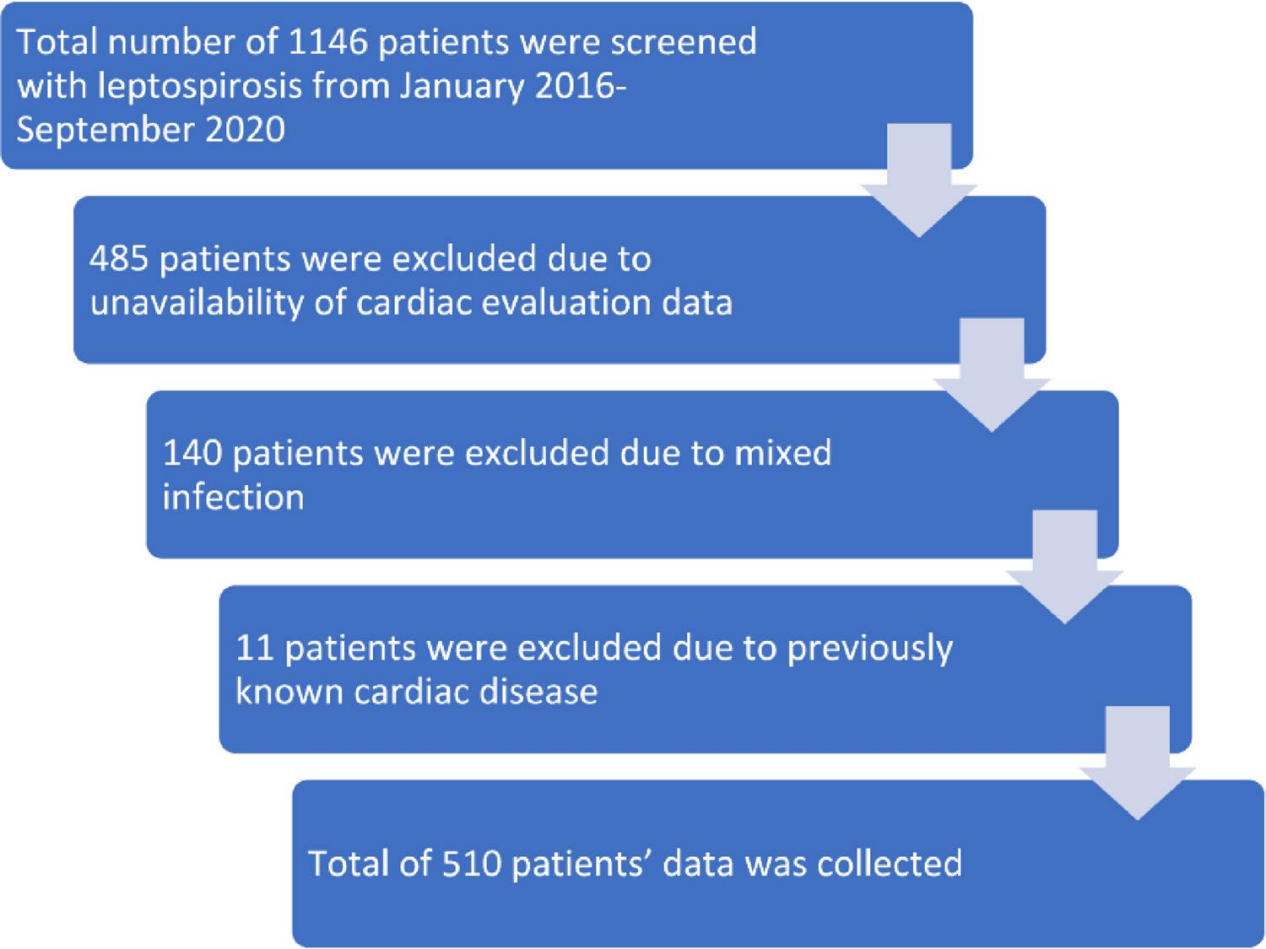
Flowchart of patient inclusion and exclusion criteria

A total of 1146 patients with clinical suspicion of leptospirosis were admitted to our hospital between January 2016 and September 2020. Of these, 485 patients lacked data on cardiac evaluation, 140 had mixed infections, and 11 had known cardiac disease. Hence, these patients were excluded from the study, and only 510 patients were included (Fig. 1).

### Baseline characteristics

The study population comprised 510 patients, including 339 men (66.5%) and 171 women (33.5%). The mean age was 48.4 ± 13.73 years. Patients were hospitalized for an average of 10.11 ± 6.13 days, and 204 individuals (40%) remained admitted for more than 10 days. Diabetes mellitus was present in 85 patients (16.7%), whereas hypertension was noted in 86 patients (16.9%). Acute respiratory distress syndrome (ARDS) occurred in 54 patients (10.6%). During the course of hospitalization, 143 patients (28.0%) developed multiorgan dysfunction syndrome (MODS). A total of 91 patients (17.8%) required hemodialysis. (Table 1)

**Table 1:** Baseline characteristics

Variable	Mean ± SD
Age (years)	48.4 ± 13.7
Male (%)	339 (66.5%)
Female (%)	171 (33.5%)
Diabetes (%)	85 (16.7%)
Hypertension (%)	86 (16.9%)
ARDS (%)	54 (10.6%)
MODS (%)	143 (28.0%)
Hemodialysis (%)	91 (17.8%)
Hospital stay (days)	10.11 ± 6.13
Hospital stay > 10 days (%)	204 (40%)

ARDS: Acute Respiratory Distress Syndrome, MODS: Multi Organ Dysfunction

### Laboratory findings

Troponin T and NT-proBNP levels were available for only 182 and 144 patients, respectively. A total of 309 patients had CK-MB data, 192 had CRP levels, and 224 had available procalcitonin data. (Table 2)

**Table 2:** Laboratory findings

Laboratory parameter	Mean ± SD
Troponin T [normal range: up to 0.02 ng/mL]	0.20 ± 0.46
CK-MB [normal range:5–25IU/L]	1421.14 ± 4.70
CRP [normal range:0–6 mg/L]	257.5 ± 1.384
NT-proBNP [normal range:40–125 pg/mL]	13443.07 ± 12752.28
Creatinine [normal range:0.7–1.2 mg/dL]	2.9 ± 2.420
Total WBC count [normal range:4.0–10.0 × 103/µL]	13865.25 ± 22297.0
Platelet count [normal range:150–400 × 103/µL]	109623.24 ± 121586.88
Total bilirubin [normal range:0.3–1.2 mg/dL]	5.68 ± 12.98
Direct bilirubin [normal range:0.0–0.4 mg/dL]	3.91 ± 5.21
ALT [normal range: up to 41IU/L]	90.41 ± 175.64
AST [normal range: up to40IU/L]	156.22 ± 361.68
Hemoglobin [normal range:13.0–17.0 g/dL]	12.23 ± 5.52
Procalcitonin [normal range: up to 0.05 ng/mL]	0.82 ± 0.39

CK-MB: Creatine Kinase MB, NT-proBNP: N-terminal pro-B-type natriuretic peptide, CRP: C-reactive protein, WBC: blood cell, AST: aspartate aminotransferase, ALT: alanine amino transferase

### Electrocardiogram parameters

The most common ECG findings were prolonged corrected QT interval (21.0%) and sinus tachycardia (20.8%). Other ECG findings include ST-T changes (7.6%), first-degree AV block (5.3%), atrial fibrillation (3.5%), sinus bradycardia (2.5%), conduction abnormalities (1.2%), ST segment elevation (2.4%), and features of pericarditis (1.6%) and ventricular fibrillation (0.6%). QTc prolongation was most prevalent in males (30.2%), followed by females (18.7%). (Table 3)

**Table 3:** Electrocardiographic data

Electrocardiographic parameter	Number of patients (%)/Mean ± SD
Sinus tachycardia	106 (20.8%)
Sinus bradycardia	13 (2.5%)
First-degree AV block	27 (5.3%)
Conduction abnormalities	6 (1.2%)
Atrial fibrillation	18 (3.5%)
Ventricular tachycardia	3 (0.6%)
Features of pericarditis	8 (1.6%)
ST-segment elevation	12 (2.4%)
ST–T changes	39 (7.6%)
QTc interval (mean ± SD)	447.99 ± 39.34
QTc prolongation	107 (21.0%)
QTc prolongation – male	81 (30.2%)
QTc prolongation – female	26 (18.7%)

AV Block: Atrioventricular Block, QTc: Corrected QT

### Echocardiographic parameters

The majority of patients demonstrated preserved left ventricular systolic function. Echocardiographic features suggestive of myocarditis were identified in 53 patients (10.4%). Isolated left ventricular dysfunction was observed in 21 patients (4.1%), while isolated right ventricular dysfunction was present in 20 patients (3.9%), and biventricular dysfunction was noted in 12 patients (2.4%). Significant pulmonary hypertension, defined as a right ventricular systolic pressure (RVSP) greater than 50 mmHg in the absence of right ventricular dysfunction, was detected in 10 patients (2.0%). Regional wall motion abnormalities with preserved left ventricular ejection fraction were found in 6 patients (1.2%). (Table 4)

**Table 4:** Echocardiographic parameters

Echocardiographic parameter	Mean ± SD/number (%)
EDD (mm)	45.12 ± 5.19
ESD (mm)	28.89 ± 4.66
EF (%)	63.02 ± 6.798
FS (%)	33.67 ± 4.63
RVSP (mmHg)	31.78 ± 8.70
RVSP > 50 mmHg (%)	10 (2.0%)
Isolated LV dysfunction (%)	21 (4.1%)
Isolated RV dysfunction (%)	20 (3.9%)
Biventricular dysfunction (%)	12 (2.4%)
RWMA with normal LVEF (%)	6 (1.2%)

EDD: end-diastolic dimension, ESD: end-systolic dimension, EF: ejection fraction, FS: fractional shortening, RVSP: right ventricular systolic pressure, RWMA: regional wall motion abnormality, LV: left ventricle, RV: right ventricle

### Outcome measures

The overall in-hospital mortality rate was 8%, with 41 patients dying during their hospital stay. Myocarditis was diagnosed in 53 patients, accounting for 10.4% of the study population. Isolated left ventricular (LV) dysfunction was observed in 21 patients (4.1%). Isolated right ventricular ((RV) dysfunction was seen in 20 patients (3.9%). Biventricular dysfunction involving both ventricles was present in 12 patients (2.4%). Resuscitated cardiac arrest occurred in 4 patients (0.8%). Among the arrhythmias, atrial fibrillation occurred in 18 patients (3.5%), whereas ventricular tachycardia was observed in 3 patients (0.6%) (Table 5).

**Table 5:** Outcome measures

Outcome	Number of patients (%)
All-cause in-hospital mortality	41 (8%)
Myocarditis	53 (10.4%)
Isolated LV dysfunction	21 (4.1%)
Isolated RV dysfunction	20 (3.9%)
Biventricular dysfunction	12 (2.4%)
Resuscitated cardiac arrest	4 (0.8%)
Atrial fibrillation	18 (3.5%)
Ventricular tachycardia	3 (0.6%)

LV: Left ventricle, RV: Right ventricle

Multivariate regression analysis was used to identify the independent predictors of in-hospital mortality. Increased age was independently associated with mortality. Among the electrocardiographic parameters, sinus tachycardia and QTc prolongation were identified as significant predictors. Hypertension and the need for hemodialysis were also independently associated with mortality. In contrast, sex, diabetes status, sinus bradycardia, and first-degree AV block did not significantly independently predict mortality in the adjusted model. The presence of myocarditis was found to be an independent predictor of mortality (Table 6). Patients with myocarditis had a significantly greater risk of death than those without myocarditis did. These findings highlight the strong prognostic impact of myocardial involvement in patients with leptospirosis.

On the other hand, diabetes and the need for hemodialysis were independent predictors of myocarditis leading to mortality in patients with leptospirosis.

**Table 6:** Multivariate regression analysis for the prediction of mortality

Variable	Exp (B)	95% C.I for Exp (B)	*P* value
Age	1.040	1.005–1.075	0.023
Sex	0.613	0.228–1.654	0.334
Sinus tachycardia	0.363	0.152–0.868	0.023
QTc prolongation	0.414	0.177–0.971	0.043
Diabetes	6.033	0.774–46.99	0.086
Hypertensive	8.369	1.0–70.042	0.050
Sinus bradycardia	1.446	0.163–12.835	0.741
First degree AV block	1.049	0.214–5.151	0.953
Hemodialysis	0.325	0.134–0.790	0.013
Myocarditis	3.728	1.743–7.972	0.001

AV Block: Atrioventricular Block, QTc: Corrected QT

## Discussion

Leptospirosis is a zoonotic disease that commonly occurs in tropical and subtropical regions and is transmitted through water and food contaminated with the urine of infected rats. Leptospirosis can range from mild symptoms such as fever and body pain to severe Weil’s disease, which includes jaundice, kidney failure, and bleeding and can ultimately lead to death. Cardiac manifestations in leptospirosis vary from arrhythmias such as atrial fibrillation, regional wall motion abnormalities, myocarditis, and pulmonary hypertension, eventually leading to mortality. Cardiac involvement, in the form of various ECG and echocardiographic changes, is well recognized but not often discussed. The aim of the current study was to investigate the cardiac manifestations of leptospirosis and to determine the predictors of outcomes in affected patients. Importantly, this is one of the largest retrospective studies on leptospirosis evaluating cardiac involvement, with a substantial sample size of 510 patients. By systematically assessing electrocardiographic and echocardiographic parameters, this study provides a clear and comprehensive understanding of the spectrum of cardiovascular involvement and its impact on patient outcomes.

This study comprehensively evaluated the spectrum of cardiac manifestations associated with leptospirosis and identified potential prognostic indicators among affected patients. In our study, the mean age of the participants was 48.4 ± 13.73 years. Among the 510 patients included, 66.5% (*n* = 339) were male and 33.5% (*n* = 171) were female. The observed male predominance is in line with existing literature on leptospirosis epidemiology. This is commonly attributed to increased occupational and environmental exposure among males, particularly in agricultural and outdoor [11].

Most patients maintained sinus rhythm, with sinus tachycardia and QTc prolongation being the most common ECG abnormalities. Other findings included ST–T changes, atrioventricular block, atrial fibrillation, and occasional ventricular arrhythmias. Overall, these abnormalities were largely nonspecific but indicated significant electrical cardiac involvement in leptospirosis. Similar patterns of ECG abnormalities, including sinus tachycardia, atrial fibrillation, conduction disturbances, and repolarization changes, have been widely reported in previous studies [12–15].

Echocardiographic assessment in our cohort demonstrated largely preserved left ventricular systolic function. However, myocardial involvement was noted in a subset of patients, including myocarditis, isolated ventricular dysfunction, and rare regional wall motion abnormalities. These findings suggest that subclinical cardiac involvement may occur even in the absence of overt dysfunction.

The overall in-hospital mortality was 8%. Arrhythmic complications and cardiac arrest were infrequent but clinically significant. Importantly, myocarditis emerged as a strong independent predictor of adverse outcomes, highlighting its prognostic significance.

Similarly, a study by Tharanga Fernando et al. observed echocardiographic abnormalities in 41% of patients, and clinical plus echocardiographic evidence of myocarditis was identified in a small subset; however, left ventricular systolic function remained preserved [13]. Similarly, C Rajiv et al. reported normal left ventricular function on echocardiography despite frequent electrocardiographic abnormalities, suggesting that overt LV systolic dysfunction is uncommon [16]. A case report by Steven Swarath et al. described new-onset cardiomyopathy with global hypokinesia and reduced ejection fraction in fulminant leptospirosis, highlighting that significant LV dysfunction, though rare, can occur in severe disease [17]. In another report, Victor Voon et al. demonstrated myocarditis with preserved ejection fraction and mild regional hypokinesia, supported by cardiac MRI findings [18]. Pathological studies further confirm myocardial involvement. T de Brito et al. identified interstitial myocarditis and coronary arteritis in fatal cases, indicating direct myocardial inflammation. Likewise, Kinjal Shah et al. reported myocarditis in the majority of autopsied hearts, suggesting that subclinical myocardial inflammation may be common even when systolic function appears preserved during life [7, 19]. A comprehensive review by Mitrakrishnan Rayno Navinan and Senaka Rajapakse emphasised that although echocardiographic evidence of acute LV dysfunction has not been consistently demonstrated, histopathological myocarditis is clearly present in severe cases and may contribute to adverse outcomes [10]. Furthermore Mathew et al. demonstrated that subclinical myocardial dysfunction is common in leptospirosis, with myocardial deformation imaging detecting early cardiac involvement even in the absence of overt echocardiographic abnormalities [20]. Jayathilaka et al. reported a series of patients with predominant cardiac manifestations, highlighting that myocarditis and associated arrhythmias can significantly contribute to disease severity and adverse outcomes [21]. Overall, ventricular dysfunction is uncommon but may occur in severe leptospirosis, while myocarditis, often clinically under-recognized, is supported by pathological evidence and carries prognostic significance. It is important to note that detecting probable myocarditis early through echocardiography can significantly reduce the mortality rate of patients with Leptospirosis.

### Limitations

This study did not include information on cardiovascular preventive medications such as statins, antihypertensive drugs, or antiplatelet agents. The absence of these data limits our ability to assess their potential influence on cardiac outcomes.

Patient identification was based on International Classification of Diseases (ICD) codes, which may carry a risk of misclassification. In addition, cardiac biomarkers such as troponin and NT-proBNP were not available for all patients, limiting accurate assessment of myocardial injury. Moreover, the diagnosis of myocarditis was not validated by gold-standard investigations, such as cardiac magnetic resonance imaging or endomyocardial biopsy. Accordingly, the findings should be considered indicative of probable myocarditis, which constitutes a key limitation of the study.

## Conclusions

In this large retrospective cohort of patients with leptospirosis, probable myocarditis emerged as a significant cardiac manifestation and was independently associated with adverse outcomes, particularly when accompanied by other systemic complications. Additionally, variables such as age, sex, and left ventricular systolic dysfunction were found to impact the likelihood of overall complications, including mortality and major organ involvement.

These findings emphasize the importance of early recognition of cardiac involvement in leptospirosis. Careful cardiac evaluation with electrocardiography and echocardiography should be considered in patients with suspected or confirmed leptospirosis, as timely identification of cardiac abnormalities may help improve risk stratification and patient outcomes.

## Data Availability

The data presented in this study are available from the corresponding author upon reasonable request, due to ethical restrictions on data sharing.
